# Supplementary material for: MicroRNA 148a Suppresses Tuberculous Fibrosis by Targeting NOX4 and POLDIP2
Source: Int J Mol Sci. 2022 Mar 10;23(6):2999. doi: 10.3390/ijms23062999 (PMC8954251; doi:10.3390/ijms23062999)
Supplement: Supplementary file 1 [file ijms-23-02999-s001.zip › Supplementary data/Supplementary Figures.pdf]

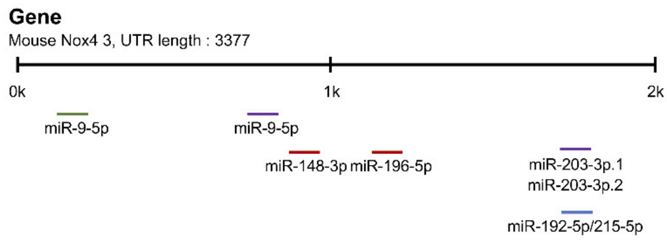

Supplementary Figure S1. Prediction of potential miRNAs that binds to 3′ UTR of mouse NOX4 gene.

A target scan was used to search for miRNAs that could interact with the 3′ UTR of the mouse NOX4 gene. miR-9-5p, miR-148-3p, miR-196-5p, miR-203-3p, and miR-192-5p/215-5p were identified.

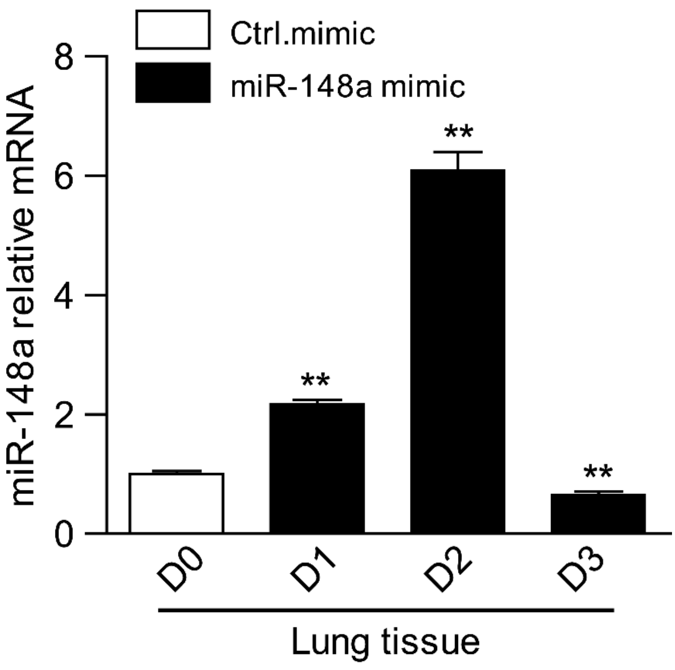

Supplementary Figure S2. miR-148a levels in mouse lung peaked on the second day from intravenous injection with miR-148a mimic (20 nmol/kg)
