# Supplementary material for: MicroRNA 148a Suppresses Tuberculous Fibrosis by Targeting NOX4 and POLDIP2
Source: Int J Mol Sci. 2022 Mar 10;23(6):2999. doi: 10.3390/ijms23062999 (PMC8954251; doi:10.3390/ijms23062999)
Supplement: Supplementary file 1 [file ijms-23-02999-s001.zip › Supplementary data/Supplementary tables.pdf]

Supplementary Table S1. The predicted sequence alignment between mouse miRNAs and NOX4 3′UTR.

ID		Alignment	Position	site
miR-9	Target (5'- 3')<br>miRNA(3'-5')	5' ....AACAGCUGUGCUAUGCCAAAGA<br>| | | | | | | | | | | | | 3' GUAUGUCGAUCUAU.......GGUUUCU	3' UTR	90-96
	Target (5'- 3')<br>miRNA(3'-5')	5' AGCUUAUCCUUAAAACCAAAGA<br>| | | | | | | 3' AGUAUGUCGAUCUAUUGGUUUCU	3' UTR	761-768
miR-196b	Target (5'- 3')<br>miRNA(3'-5')	5' UCAGAAGGAAAAUUUACUACCUU<br>| | | | | | | 3' GGGUUGUUGUCCUUUGAUGGAU	3' UTR	1048-1054
miR-148a	Target (5'- 3')<br>miRNA(3'-5')	5' CACUAAUAUUAUUUUUGCACUGU<br>| | | | | | | 3' UGUUUCAAGACAUCACGUGACU	3' UTR	941-947
miR-215	Target (5'- 3')<br>miRNA(3'-5')	5' ACAUUUCAAAAAUUCAGGUCAAA<br>| | | | | | | | | | | 3' CAGACAGUUUAGUA - UCCAGUA	3' UTR	1565-1571
miR-203	Target (5'- 3')<br>miRNA(3'-5')		CDS	-

Supplementary Table S2. The baseline characteristics of patients with transudate and tuberculous pleural effusion

Group	Subject	Age	Sex	Sputum AFB culture	PE AFB culture	PE ADA	PE/Serum protein	PE/Serum albumin
TB-PE	TB-PE 1	75	M	Negative	Positive	6.2	1	0.74
	TB-PE 2	21	M	Negative	Positive	6.7	0.61	0.67
	TB-PE 3	92	F	Positive	Negative	30	0.78	0.75
Transudate	T1	84	M	Negative	Negative	36.9	0.41	0.36
	T2	80	F	Negative	Negative	<4.0	0.26	0.21
	T3	92	F	Negative	Negative	7	0.39	0.44

PE, pleural effusion; AFB, acid-fast bacilli; ADA, adenosine deaminase; TB-PE, tuberculous pleural effusion; LDH, lactate dehydrogenase; CRP, C-reactive protein
